# Current knowledge of TNF-α monoclonal antibody infliximab in treating Kawasaki disease: a comprehensive review

**DOI:** 10.3389/fimmu.2023.1237670

**Published:** 2023-10-23

**Authors:** Jiaying Chen, Jian Liao, Lupeng Xiang, Shilong Zhang, Yajing Yan

**Affiliations:** ^1^ Department of Pediatrics, Taizhou Central Hospital (Taizhou University Hospital), Taizhou, Zhejiang, China; ^2^ Department of Nephrology, Jiaxing Hospital of Traditional Chinese Medicine, Jiaxing, Zhejiang, China; ^3^ Taizhou University Medical School, Taizhou, Zhejiang, China; ^4^ Clinical Medical College, Zhejiang Chinese Medical University, Hangzhou, Zhejiang, China; ^5^ Health Management Center, Taizhou Central Hospital (Taizhou University Hospital), Taizhou, Zhejiang, China

**Keywords:** Kawasaki disease, infliximab, treatment, adverse effect, TNF-α

## Abstract

Kawasaki disease (KD), an autoinflammatory disease primarily affecting young children, characterized by consisting of acute systemic vasculitis and coronary artery involvement in severe cases. Intravenous immunoglobulin gamma (IVIG) combined with aspirin therapy is the first-line regimen for the prevention of coronary aneurysms in the acute phase of KD. The etiology and pathogenesis of KD are unclear, but its incidence is increasing gradually, especially in the cases of IVIG-naïve KD and refractory KD. Conventional therapies for refractory KD have unsatisfactory results. At present, infliximab (IFX), a human-murine chimeric monoclonal antibody that specifically blocks tumor necrosis factor-α (TNF-α), has made great progress in the treatment of KD. This review revealed that IFX infusion (5 mg/kg) could effectively modulate fever, reduce inflammation, improve arthritis, diminish the number of plasma exchange, decrease hospitalizations, and prevent the progression of coronary artery lesions. The adverse effects of IFX administration included skin rash, arthritis, respiratory disease, infusion reaction, hepatomegaly, and vaccination-associated complications. But the incidence of these adverse effects is low. The clear optimal application protocol of the application of IFX for either initial combination therapy or salvage therapy in KD is still under investigation. In addition, there are no effective biomarkers to predict IFX resistance. Further multicenter trials with large sample size and long-term follow-up are still needed to validate the clinical efficacy and safety of IFX for IVIG-resistant KD or refractory KD.

## 1 Introduction

Kawasaki disease (KD), also known as cutaneous mucosal lymph node syndrome, was first reported by Dr. Tomisaku Kawasaki in Japan in 1967 (1). KD is a systemic inflammatory vasculitis syndrome and often occurs in children who under 5-year-old. KD occurs throughout the year, and the peak is detected during the winter–spring seasons. At present, the global incidence of KD is increasing gradually (2). According to the epidemiological data, KD is more prevalent in Japan (3) and Korea (4) when compared to the Western countries (5, 6). Of note, the incidence of KD in children under 5 years of age is low in Western countries, while the incidence in East Asian countries continues to elevate. The most serious complication among patients with KD is the cardiovascular disease, i.e., coronary artery lesions (CAL), cardiac lesions, and peripheral arterial lesions. CAL is the most common complication which can lead to myocardial ischemia, myocardial infarction, and even death, seriously threating to children’s health and life (7). The American Heart Association’s guidelines for the diagnosis of KD show that 15% to 25% of untreated children will develop coronary artery aneurysms (CAA) or coronary artery dilation (CAD), which have become the leading cause of acquired heart disease in children. The first-line treatment for the acute phase of KD is to resolve the intense inflammation in the acute phase as early and to minimize the incidence of CAL. Currently, intravenous immunoglobulin gamma (IVIG) combined oral aspirin serves as the first-line treatment option for KD, which can reduce the risk of CAA from 25% to 5% (8). The administration of this therapeutic strategy is IVIG either as a single infusion of 2 g/kg over 10-12 hours combined with aspirin (100 mg/kg daily through the 14th day of illness, then 3 to 5 mg/kg daily) (9–11). However, 10-15% of children with KD still do not respond to IVIG regimens (12). Moreover, the probability of CAL is significantly higher in IVIG-refractory children than those children with IVIG-sensitive response (13). Recent data from Japan suggest that the IVIG resistance rate has increased from 7% in 2003 to 23% in 2014, with a concomitant increase in the incidence of CAA (14). Therefore, how to effectively treat KD (especially IVIG-refractory KD) remains a hotspot in clinical research. There is no unified treatment pattern being followed for the IVIG-refractory children. The common treatments include the second dose of IVIG, glucocorticoids, and infliximab (IFX) (7).

The host’s immune system is a major determinant in the pathogenesis of KD (15). Clinically, biological agents are recommended currently for treating IVIG-refractory children with KD (16). IFX is a human-murine chimeric monoclonal antibody that specifically blocks tumor necrosis factor-α (TNF-α) via binding to TNF-α with high affinity and inhibiting TNF-α from binding to its receptor, and therefore exhibits anti-inflammatory effects. IFX is indicated for rheumatoid arthritis, ankylosing spondylitis, psoriatic arthritis, and Crohn’s disease (17). Besides, it is the first anti-TNF-α monoclonal antibody that has been validated for the treatments in pediatric patients (18). At present, an increasing number of studies have found that IFX might exhibit a better therapeutic profile for patients who failed initial IVIG monotherapy. According to the American Heart Association (AHA) 2017 Scientific Statement, three most common second-line therapies are recommended for treating KD, including a second dose of IVIG, intravenous methylprednisolone or a single dose of IFX infusion (7). For these IVIG-resistant KD patients, patients receiving IFX as second therapy had a higher response rate, faster resolution of fever, lower incidence of CAAs, and fewer days of hospitalization than those who received only a second IVIG dose (19–22). For example, the data from the United States (2006-2015 yr) showed that the response rates in KD patients treated with IFX, steroids, and a second IVIG dose were 87.9%, 83.0%, and 73.3%, respectively (P < 0.001) (19). However, despite its promising therapeutic effects, the rates of IFX application in patients with refractory KD (or received the second-line therapies) was low in some geographic regions (20). In this study, we conducted a comprehensive review on the applications of IFX in KD based on the current evidence.

## 2 The mechanisms of action of IFX

In the acute phase of KD, plasma levels of TNF-α were significantly increased and were higher in children with combined CAL than in those without CAL (7). As a result, blocking TNF-α is an effective intervention in regulating inflammation in KD. Mechanistically, TNF-α directly induces vascular endothelial cells to express intercellular adhesion molecule-1 (ICAM-1), monocyte chemoattractant protein-1 (MCR-1), and promote inflammatory cell infiltration (i.e., neutrophils and monocytes, thus aggravating the inflammatory injury of the blood vessels (23). In contrast, IFX, a monoclonal antibody that specifically blocks TNF-α, is confirmed to exert an excellent anti-inflammatory effect. In the previous studies, Maury et al. (24) and Lang et al. (25) demonstrated that high TNF-α expression were observed in the blood of patients with KD. Consistently, in a subsequent study in Japan, Matsubara et al. (26) found a similar result and showed a correlation between TNF-α and CAA complications. A study (27) in Canada revealed that specific blockade of TNF-α prevented the formation of coronary arteritis and coronary aneurysms in a mouse KD model. Oharaseki et al. (28) found that TNF-α antagonists such as IFX could reduce the adhesion of neutrophils to vascular endothelial cells in the acute phase, thus attenuated the inflammation of the arterial vascular endothelium.

IFX administration was found to reduce the serum levels of interleukin-6 (IL-6), soluble tumor necrosis factor-alpha receptor I (TNF-R I) and other inflammatory levels, especially in children with KD complicated by CAL (29). However, IFX could not reduce the levels of vascular endothelial growth factor (VEGF) and myeloid-associated proteins. This evidence suggested that IFX might be effective in suppressing systemic inflammation but not completely blocking local vasculitis in KD. In addition to a significant decrease of multiple factors induced by the stimulation of the TNF-α pathway in KD, IFX treatment also controlled the inflammatory cytokine pathways of IL-1 and IL-6 and reduced the inflammatory cytokine pathways of peptidase inhibitor-3 (PI-3), matrix metalloproteinase 8 (MMP-8), chemokine receptor-2 (CCR-2) and pentraxin-3 (PTX-3) transcript levels (30). Besides, IFX can also inhibit the elastase secretion between neutrophils (31).

## 3 Data sources and strategy of search

In this study, four common electronic databases, e.g. MEDLINE (PubMed), EMBASE databases, Cochrane Library databases, and Google Scholar, were systematically searched to find the eligible studies. The timeframe spanned from the inception of these databases to June 1, 2023. Only human participant research that acknowledged utilizing the English language was considered to be eligible. The following search terms were used in different combinations in PubMed: ((((“Mucocutaneous Lymph Node Syndrome”[Mesh]) OR (Kawasaki Syndrome)) OR (Lymph Node Syndrome, Mucocutaneous)) OR (Kawasaki Disease)) AND ((((((((((((“Infliximab”[Mesh]) OR (MAb cA2)) OR (Monoclonal Antibody cA2)) OR (Antibody cA2, Monoclonal)) OR (cA2, Monoclonal Antibody)) OR (Infliximab-dyyb)) OR (Infliximab dyyb)) OR (Inflectra)) OR (Remicade)) OR (Infliximab-abda)) OR (Infliximab abda)) OR (Renflexis)). Also, reference lists were manually searched for detecting more eligible studies. As shown in **Supplementary Figure 1**, in total, 652 studies were found in the four databases during the initial search. Finally, 24 studies were considered eligible after removing duplicates and those studies failing to meet the inclusion criteria.

## 4 Eligibility criteria and data extractions

The criteria for the selection of the eligible studies included: 1) participants (articles including a population of KD patients; 2) the design of the studies (any study design); 3) the intervention (with the treatment of IFX); 4) comparative group (with or without a control group); 5) outcomes (treatment efficacy and adverse effects). We extracted those data from the eligible studies, including the first author, publication year, geographic distribution, sample size, IFX administration, treatment outcomes or main findings, and the adverse events under IFX treatment.

## 5 IFX for the treatment of KD complications

### 5.1 Modulation of fever and inflammation

#### 5.1.1 Initial treatment

According to the current evidence, IFX is effective in initial treatment, refractory treatment, and combination therapy for KD. IVIG therapy is an effective method to treat autoimmune and immune associated diseases, including KD. Some investigators applied IFX in the initial treatment of KD. In 2009, Hirono et al. (29) studied 43 patients with KD, including 18 IVIG-responsive, 14 IVIG-nonresponsive, and 11 patients initially treated with IFX. Ten patients received IFX (5 mg/kg) as a single infusion and one patient received twice 5 mg/kg of IFX. Among these patients treated with IFX, 8 children had significant fever remission, of which six had fever that resolved immediately after IFX treatment, one patient resolved within 24 hours and one within 48 hours, and other symptoms also disappeared. Of note, four patients had concomitant CAL even after IFX treatment, which might be correlated to the abnormal coronary arteries with transient dilation had been documented by echocardiography before IFX treatment. Inflammatory factors CRP and IL-6 were significantly decreased after IFX treatment, suggesting a positive role for IFX as remedial therapy or initial intensive therapy in KD.

Shirley et al. (32) reported a case of a 3-year-old male with KD who developed the diffuse urticaria and facial swelling after IVIG infusion. This child was treated with IFX (5mg/kg) and subsequently given methylprednisolone 30 mg/(kg-d) for 3 days, resulting in his fever symptom quickly alleviated. No adverse effects were observed during the initial treatment and subsequent follow-up. In this case report, IFX provided a new approach to the initial treatment of KD, but the efficacy of IFX alone could not be evaluated because of the concomitant use of high-dose methylprednisolone therapy, so its clinical significance was relatively limited.

#### 5.1.2 Refractory treatment

Refractory KD management is a thorny clinical problem. IFX showed potent effect on KD patients with IVIG failure or refractory KD. In 2004, Weiss et al. (18) performed a case report of a 3-year-old child with refractory KD who had recurrent fever and progression of coronary aneurysm despite eight doses of IVIG and eight times of high-dose methylprednisolone therapy. Besides, this patient continued to present with elevation of erythrocyte sedimentation rate, platelet count, and liver enzymes, accompanied by decreased hemoglobin and albumin. After the first infusion of IFX, the child had no further fever. In 2005, Burns et al. (33) reported IFX as a secondary treatment for IVIG-resistant patients after IVIG administration. Seventeen subjects treated with IFX, 14 children remarkably ceased fever. Fifteen children received a single dose of IFX 5 mg/kg and the remaining two children with arthritic complications received a single dose of IFX 10 mg/kg, the arthritis symptoms were significantly relieved within 12 hours after IFX infusion. CRP was elevated in most of the patient (16/17, 94%) before IFX infusion and decreased after infusion of IFX in 10 patients who were remeasured within 48 hours of treatment. Four patients had transient dilatation that disappeared after IFX infusion. Fifteen patients were followed up for 6 to 26 months without significant complications after IFX treatment. This study demonstrated that patients with dilated coronary arteries might restore after IFX infusion.

According to the published data, IFX could serve as one of the treatments after IVIG failure. In 2016, Youn et al. (34) reported that 43 children with KD resistant to initial IVIG were randomly assigned to receive a second dose of IVIG (n=32) or IFX (n=11). The results showed that 21 children (65.6%) responded to IVIG retreatment and 10 children (90.9%) responded to IFX, suggesting that the need for further treatments in the IFX group was less than the IVIG group. Retreatment with IFX for the first time might improve the efficiencies of treatment. The mean duration of fever was 6 hours in the IFX retreatment group and 17 hours in the IVIG retreatment group, while the number of children who resolved their fever within 24 hours was 9/11 (81.8%) and 18/32 (56.3%) in IFX and IVIG group, respectively. The mean number of hospital days was 8 days in the IFX group and 10 days in the IVIG group, indicating that IFX treatment shortened the duration of fever and hospitalization. One patient in IFX group and four IVIG patients in the IFX group developed CAL complications. All these five patients required a second retreatment and had no coronary abnormalities on echocardiography early in the disease. The remaining 31 patients did not present with CAL, which might be associated with the effective response to the first treatment of IVIG or IFX. One child in the IFX group developed a rash during the infusion, while five children in the IVIG group developed a febrile infusion reaction that required suspension of the infusion for relief. This study shows that initial retreatment with IFX did not reduce the incidence of CAL (reduction of coronary artery abnormalities) but reduced further treatment compared to IVIG, i.e. IFX may be effective in reducing inflammatory markers but less effective in inhibiting vasculitis itself, suggesting IFX as a retreatment option after unsuccessful initial IVIG therapy in KD patients. In line with Youn et al.’s study, Son et al. (22) reported that patients with IVIG-resistant KD who received IFX treatment had faster resolution of fever and fewer days of hospitalization. However, IFX could not improve coronary artery outcomes. No AEs related to IFX treatment were detected during the follow-up.

Subjects with KD often suffer from concomitant comorbidities. Ohnishi et al. (35) reported a case of a 14-month-old boy diagnosed with KD combined with Yersinia pestis small intestinal colitis. This child presented with non-bloody watery diarrhea seven times a day without vomiting or abdominal distention and without pressure pain. All symptoms disappeared except fever and diarrhea after twice IVIG treatments. On day 12 of the course of the disease, the fever subsided with the administration of IFX 5 mg/kg, but the diarrhea continued. On day 15, this patient developed fever again, then received a third administration of IVIG and oral cyclosporine A 4 mg/kg with cefotaxime sodium injection for anti-infective treatment. For 5 days under these treatments, no abnormality was seen on follow-up cardiac ultrasound. This finding indicated that small bowel colitis might mimic or be involved in the pathogenesis of KD, and therefore treatment of KD along with antimicrobial therapy is required. Immunosuppression and monoclonal antibody therapy are currently considered to be additional therapies for IVIG-resistant KD (7).

Song et al. (36) retrospectively analyzed 16 cases of refractory KD, 14 of which were children with persistent or recurrent fever and 2 with fever that subsided after symptomatic management with supportive therapy. Sixteen children had elevated CRP before IFX treatment, which decreased after treatment in all 14 children for whom results were available. Coronary artery (CA) abnormalities (ranging from mild CA dilatation to aneurysms) were detected on echocardiography before IFX treatment in 15 patients. Three of them had transient mild CA dilatation that resolved after IFX injection, 9 had CAA, which returned to normal in 4 patients after treatment, 3 had persistent mild dilatation, 2 had persistent aneurysms at echocardiographic follow-up. Among the three patients who were not followed up, two of them had CAA and one had mild CA dilatation. This study suggested that CA dilatation stopped or at least slowed down after IFX treatment. It was possible that there was no significant additional progression of CA lesions after IFX treatment and that IFX treatment of refractory KD might have prevented the rapidly progressive CA expansion. It is proposed that early administration of IFX should be considered if CAL progresses rapidly in the presence of other treatments (including methylprednisolone). In those patients combined with persistent arthritis, all of whom had received two or more doses of IVIG or IVIG combined with pulsed methylprednisolone. Thirteen of these children had fever resolution within 12 hours after IFX treatment, including two children with combined arthritis. The arthritis was significantly relieved after IFX treatment. One of the children developed acute hepatitis during IFX treatment and stone cholecystitis after 4 months of treatment. Fifteen of the sixteen patients (15/16, 94%) did not have infusion reactions or complications from IFX administration. Of note, Dogra et al. (37) reported that an 18-mo-old girl who presented with features of incomplete KD and was refractory to IVIG and IFX. Fortunately, this patient was response to pulse intravenous methylprednisolone therapy. This study indicated pulse intravenous methylprednisolone therapy might be an option for the patients who failed to first-line and second-line treatment.

#### 5.1.3 Combination therapy

Combination therapy also plays an important role in treating KD. In 2012, Servel et al. (38) reported a case of KD with hemophagocytic syndrome that was resistant to treatment after treating with twice IVIG and three times of corticosteroid combinations. Interestingly, the patient’s condition returned to normal after a single treatment with IFX. Sivakumar et al. (39) reported a 7-month-old male child with KD complicated by extensive coronary artery involvement and coronary thrombosis. Multiple doses of IVIG and steroid therapy failed to control the inflammation, recurrent fever persisted after IFX infusion. In this case, the patient took a long-term oral steroid medication and combined with the platelet glycoprotein 2b/3a receptor blocker “abciximab”, which turned out to be the reduction in thrombus size and coronary aneurysm size, followed by fever regression.

Tremoulet et al. (11) conducted a randomized, double-blind, placebo-controlled phase 3 clinical trial in which 196 children with KD. The patients were randomized to 2 groups and given IFX 5 mg/kg and a placebo control, both of which were subsequently given IVIG combined with aspirin in a standard regimen. The results showed that 11 children in both the IFX-treated and control groups did not respond to treatment, and there was no significant difference in the incidence of CAL between the two groups. However, the duration of fever was shorter in the IFX group (median of 1 day for IFX versus 2 days for placebo, P<0-0001). The inflammatory markers including C-reactive protein, absolute neutrophil count, and erythrocyte sedimentation rate decreased rapidly after IFX treatment. More clinically significant, the Z-score of the left anterior descending branch of the coronary artery was significantly decreased in the IFX group than the control group at 2 weeks after treatment. These results suggested that the standard treatment regimen given in combination with IFX in the initial treatment of KD could further reduce inflammation and decrease the occurrence of coronary artery lesions. However, this regimen cannot improve IVIG resistance, thus its efficacy, especially on coronary artery lesions, still needs further verification. Pilania et al. (40) reported that four children with KD combined with macrophage activation syndrome (MAS) received IVIG as first-line therapy and then treated with IFX. The outcomes turned out to be no recurrences of MAS were detected in the four patients under IFX treatment, and none of them had any short-term adverse effects.

### 5.2 Treatment of arthritis

Arthritis can be seen in the acute phase of KD. In 2022, Matsubara et al. (41) reported a case of refractory KD-related delayed arthritis in a 4-year-old girl who was diagnosed with KD on day 5 of the disease and initially had no complications of CAL. After several times of IVIG combined with oral prednisolone (PSL) 2 mg/kg and intravenous ulinastatin, the fever reappeared during hormone tapering (day 17 of the course of the disease). On day 18, the patient developed pain in the right and left hip, knee and ankle joints. She was diagnosed with late-onset arthritis associated with refractory KD. After treatment with IFX (5 mg/kg), the child’s fever and arthralgia were significantly relieved, and the coronary arteries did not change significantly on review. The child’s arthritis did not recur and the coronary arteries returned to normal after 6 months of follow-up. In this study, delayed arthritis that was ineffective to treatment with IVIG, steroids, ulinastatin and cyclosporine, and was accompanied by worsening CAL. This observation suggested that additional IFX therapy should be considered for arthritis associated with refractory KD.

### 5.3 Diminished the number of plasma exchange

Shimada and Matsuoka et al. (42) investigated the effect of IFX (5 mg/kg) given before plasma exchange (PE) treatment on patients with KD. KD patients who received IFX before receiving PE were in the IFX group and those who did not receive IFX were in the non-IFX group, and the CRP levels were lower in the IFX group. After treatment, CRP, tumor necrosis factor-α (TNF-α), granulocyte colony-stimulating factor (GCSF), and monocyte chemotactic protein-1 (MCP) were significantly lower in the IFX group than in the non-IFX group. The total number of PE treatments was less than in the non-IFX group. The reason for this finding might be correlated to that TNF-α was a key inflammatory cytokine in KD, while IFX was an anti-TNF-α monoclonal antibody that binds to TNF-α. PE suppressed inflammation by directly removing inflammatory cytokines from plasma Both therapies have been used to treat IVIG-resistant patients. Although IFX treatment was not clinically sufficient to achieve remission, IFX might have reduced circulating levels of TNFα, and thus subsequent PE rapidly controlled inflammation. Thus, the application of IFX for KD prior to PE treatment resulted in a faster reduction of inflammatory markers, which improved clinical symptoms and reduced the number of treatments for PE. There was also no significant difference in the coronary z-score 1 month after the onset of KD between the two groups. Further, there was no significant difference in the incidence of complications associated with PE between the two groups. In line with the above findings, Ebato et al. (43) and Sonoda et al. (44) also reported the clinical efficacy and safety of IFX used together with PE, showing that the use of IFX before receiving PE reduced the total duration of PE, decreased the number of catheter punctures and sedation, and reduced physical restraint.

Miyata et al. (45) conducted a prospective observational study of RAISE (Prospective Observational Study on Stratified Treatment With Immunoglobulin Plus Steroid Efficacy for Kawasaki Disease Prospective Observational Study on Stratified Treatment With Immunoglobulin Plus Steroid for Kawasaki Disease) and found that young age at fever, large maximal Z-score of coronary internal diameter at baseline, and unresponsiveness to IVIG and prednisolone therapy were important risk factors for the development of CAA. More intensive or adjuvant therapy with drugs such as IFX should be considered for these KD patients with high-risk factors in order to reduce the development of CAA. It is proposed that baseline total bilirubin values enable the identification of non-responders at the time of diagnosis.

In a multicenter, prospective, open-label, single cohort, observational study, Miura et al. (46) recruited 208 patients with KD who had failed conventional therapy and received a single dose of 5 mg/kg IFX. The authors found that the fever regression rate was 77.4% within 48 hours after IFX infusion. The incidence and severity of coronary artery lesions did not change significantly after IFX administration. Adverse drug reactions and serious adverse drug reactions occurred in 12.4% and 3.1% of patients, respectively. A significantly higher incidence of serious adverse reactions was found in infants than in other age groups. No live vaccination-associated infections were observed.

Real-world studies have found that a single infusion of IFX was well tolerated and effective for fever in patients with acute refractory KD. Dionne et al. (47) found that in children with KD complicated by CAA at diagnosis, initial treatment with superimposed IFX or hormones delayed the progression of CAA compared with IVIG alone.

## 6 Efficacy of IFX treatment

The efficacy of IFX treatment for KD was variable among different studies. Han et al. (48) studied the therapeutic effects of conventional IVIG therapy versus IVIG combined with IFX therapy in KD. They found that the fever, CRP, WBC, and TNF-α decreased earlier and more significantly in the combined group than in the IVIG group. IFX treatment significantly reduced the incidence of CAA in KD patients compared with conventional IVIG therapy. Several subsequent studies (11, 19) have shown that IFX induced lower levels of inflammatory markers, shorter duration of fever, reduced length of stay (LOS), and a lower likelihood that patients could require further treatment. These advantages resulted in lower healthcare costs worldwide. Burns et al. (49) conducted a two-center retrospective study of intravenous immunoglobulin-resistant disease with a second intravenous immunoglobulin resulted in faster fever relief and shorter length of stay compared to IVIG. Johnson et al. (50) reported a cost comparison between IFX and intravenous IVIG for refractory KD. They found that IFX was more cost-saving than IVIG treatment. IVIG was easy to face the national shortage. Thus, cost savings can be achieved by giving IFX replacement therapy when more than a second dose of IVIG is used in children with refractory KD. Hur et al. (21) conducted a study to examine the efficacy of IFX in treating patients with IVIG-resistant KD in Korea. This study showed that early use of IFX after IVIG treatment might cause a shorter duration of fever, less days of hospitalization, and the prevention of CA complications in patients with IVIG-resistant KD.

Nagatomo et al. (8) showed that the cumulative persistence of CAA at 2, 4, and 6 years was 24%, 24%, and 24% in the IFX group compared with 67%, 52%, and 33% in the non-IFX group, respectively. In patients with moderate and large CAA, the cumulative persistence at 2, 4, and 6 years was 33%, 33%, and 33% in the IFX group compared with 77%, 51%, and 48% in the non-IFX group, respectively. IFX not only inhibited the inflammatory response to CAA development, but might also assist in achieving vascular remodeling after early CAA regression. A later study by Suzuki et al. (51) showed that early administration of IFX might reduce the incidence of significant CAA in patients with IVIG-resistant KD. Jone et al. (52) conducted a study for investigating children diagnosed with KD with CAL at the time of presentation. They found that IFX combined with IVIG as initial therapy reduced the need for additional therapy in children with KD complicated by CAL. Therefore, IFX might reduce the number of patients with IVIG-resistant KD. IVIG plus IFX group had only 1 infusion reaction occurred, while the IVIG group had more adverse events. No patient in either group developed serious bacterial or viral infections within 3 months after treatment.

IFX for refractory KD is generally well tolerated and safe, with minimal risk of infusion reactions or serious adverse events (19). Li et al. (53) found that adding IFX therapy to standard treatment was associated with improved treatment response, prevention of progressive CAA, and no increase in AEs. In addition, this study indicated that a beneficial effect of IFX could be found to set it as an initial treatment for high-risk patients. However, the treatment of IFX remains a controversial issue in the long-term follow-up of CAA, so more studies and trials are needed to evaluate the importance of IFX in KD patients with CAA (53). Miura et al. (46) performed in a prospective multicenter study and found that a single infusion of IFX was well tolerated in patients with treatment-refractory KD on conventional therapy and was effective in relieving fever. No significant change was found in the incidence or severity of coronary artery lesions after IFX infusion compared with controls. This finding was consistent with the safety profile obtained in clinical studies in similar groups of KD patients treated with IFX in Japan (1, 16), the United States (11), and Korea (34). Tremoulet et al. (11) found IFX was safe and well tolerated in a phase III clinical trial of IFX 5 mg/kg adjuvant therapy, even in infants younger than 6 months of age.

## 7 Adverse effects

The common adverse effects of IFX include skin rash, arthritis, respiratory disease, infusion reaction, hepatomegaly, and vaccination-associated complications. Complications of IFX administration include reactivation of latent tuberculosis, histoplasmosis, increased risk of bacterial sepsis and lymphoma, production of antinuclear antibodies, and heart failure. Since the routine administration of IFX in treating children with KD is single infusion (children with other diseases always require multiple IFX treatments), the incidence of reported adverse reactions to IFX is low (46, 54, 55).

### 7.1 Arthritis and skin rash

Mitsui et al. (56) reported a case of a 7-year-old child with KD who developed psoriasis-like rash and arthritis after treatment with IFX. On the day 4 of illness onset, the child was treated with IVIG (2 g/kg), intravenous methylprednisolone (IVMP, 30 mg/kg/day), oral prednisolone (PSL, 2 mg/kg/day), but had persistent fever. On day 8, IFX (5 mg/kg) was given, the fever and other symptoms improved immediately. After taking PSL (1 mg/kg/day) on day 34, his joint symptoms were rapidly reduced and his psoriasis-like rash gradually improved. Since then, the PSL dose was gradually reduced over 6 months. This is consistent with the finding reported by Haddock et al. (57). Youn et al. (34) reported that one IFX-treated subject did develop a rash during the infusion.

Without IFX treatment, Guleria et al. (58) showed that 29 (72.5%), 8 (20%), and 3 (8.6%) children developed arthritis in acute, subacute, and convalescent phases of KD, respectively. With the treatment of IFX, Sonoda et al. (44) reported that 10.5% of KD patients presented with arthritis within 2-3 weeks after IFX therapy. In the real-world SAKURA study (46), the incidence of arthritis after IFX treatment was recorded at 2/291 (0.7%). In the cases with IFX treatment other than KD, arthritis flare has been reported as a paradoxical adverse reaction after IFX administration for rheumatoid arthritis (59) and psoriatic arthritis (60). But for treating of KD, IFX is administrated with a single infusion. Therefore, the frequency of arthritis among patients with refractory KD receiving IFX is lower than in other diseases due to KD patients did not receive IFX administration repeatedly. Based on the current evidence, whether arthritis is a complication of IFX or part of the disease of KD, which is still confused and warrants further investigation. Since arthritis is one of the symptoms of KD, the clinicians should make a determination as to whether the complaint was related to the IFX if this complaint predated IFX infusion.

### 7.2 Upper airway complications

In a phase 3 multicenter trial, Mori et al. (16) showed that four cases of concomitant nasopharyngitis, three cases of upper respiratory tract infection, and two cases of upper respiratory tract inflammation in the IFX-treated group. These patients had increased anti-double-stranded DNA (anti-dsDNA) antibodies but decreased to normal levels during follow-up. No lupus-like syndrome was observed in any of the patients. Singh et al. (61) reported that one patient (1/23) developed an acute febrile illness two weeks after IFX treatment (5–7 mg/kg given intravenously) which responded well to anti microbials.

### 7.3 Liver complications

Burn and colleagues (49) reported transient hepatomegaly in five subjects during the second week of IFX treatment, while only one subject exhibited transient hepatomegaly during the first week of IVIG treatment. Subsequently, Son et al. (22) also reported a 19% incidence of transient hepatomegaly in patients treated with IFX (6/31) compared to 1.5% in the controls. Of note, anti-TNF- α drugs have also been used to treat severe stages of liver disease, such as alcoholic hepatitis (AH), nonalcoholic fatty liver disease (NAFLD), refractory autoimmune hepatitis (AIH), and primary biliary cholangitis (PBC), which were contradicting with IFX -associated hepatotoxicity (62).

### 7.4 Complications related to vaccination

Other complications of IFX administration included reactivation of latent tuberculosis and an increased risk of bacterial sepsis (63). IFX has been reported to induce central nervous system demyelination episodes during anti-TNF-α therapy or to lead to fatal outcomes due to BCG vaccination (64, 65). In Japan, an interval of at least 6 months is recommended for the application of IFX in children who have received live vaccines (especially BCG) before the diagnosis of KD. Besides, IFX treatment should be used at least 3 months of interval from other live attenuated vaccinations, due to a shorter interval may lead to a higher risk of infection (46). Song et al. (36) reported that 38 children who received live attenuated vaccine within 90 days prior to IFX application. Furuta et al. (54) reported 6 children who received BCG vaccine between 14 d and 8 months prior to IFX application. Both Song et al. and Furuta et al.’s studies demonstrated that none of whom had complications with tuberculosis or other serious infections. Burns et al. (49) and Tremoulet et al. (11) reported that patients with KD could safely receive IFX treatment even if they had recently received live virus vaccine. At present, relevant data on the time interval between live vaccination and IFX use and the risk of live vaccination-related (especially BCG) infections are currently limited and require further study.

### 7.5 Infusion reaction

Jone et al. (52) reported that the incidence of infusion reactions in the IFX combined with IVIG treatment group was significantly lower than that in the IVIG treatment group. Only one infusion reaction occurred after IVIG combined with IFX for KD. But this adverse effect might be partially caused by IVIG due to this observation was derived from IFX combined with IVIG treatment. Currently, a direct correlation with IFX-mediated infusion reaction could not be established due to a combination therapy is commonly applied at this time. As a result, further studies are still needed to validate this adverse effect.

Of note, IFX can effectively treat various autoimmune diseases, including KD, rheumatoid arthritis, Crohn’s disease, and psoriasis. KD often occurs in children who under 5-year-old, while rheumatoid arthritis, Crohn’s disease, and psoriasis commonly occur in adolescents, young adults, and middle-aged adults. Therefore, there might be some differences of side effects of IFX between children and adults. Due to IFX is administered only once in most patients with KD, its administration may cause fewer complications in KD patients than in patients with other autoimmune diseases, where it is administered repeatedly (36).

## 8 Roles of TNF-α antagonist (i.e., IFX) in KD patients with SARS-CoV-2 and patients with MIS-C

Current evidence suggested that the coronavirus (SARS-CoV-2) might have some impacts on children with Kawasaki disease. On the other hand, several studies have shown that some children with coronavirus infections developed symptoms similar to KD, including high fever, rash, conjunctivitis, oral mucositis, and red, swollen, and pain and swelling in the limbs (66). This condition is known as COVID-19-associated multisystem inflammatory syndrome (MIS-C). However, as compared to patients with KD, MIS-C patients typically have the following characteristics (67). First, patients with MIS-C are older than KD patients. Second, patients with MIS-C are susceptible to severe systemic inflammatory response, i.e., high fever, rash, and inflamed mucosa. Third, MIS-C can be associated with cardiac injury, i.e., myocarditis, dilated or abnormal coronary arteries, and heart failure. KD is commonly a self-limiting disease, while MIS-C may require longer treatment and care. There may be a connection between KD and MIS-C. As reported, angiotensin converting enzyme (ACE)-2 receptor is downregulated by the SARS-COV through the spike protein of SARS-CoV via a process that is tightly coupled with TNF-α production (68). Since ACE2 has been described to exhibit anti-inflammatory, anti-fibrotic, and anti-proliferative function, ACE2 plays an important role in KD development. Amirfakhryan (68) demonstrated that KD children with genetically low-expression of ACE2 receptor might aggravate inflammation and elevate TNF- α production. Due to IFX is a TNF-α antagonist, patients with MIS-C might be successfully treated with IFX (69). For the KD patients with SARS-CoV-2 infection, Roh et al. (70) reported that post-COVID KD showing a stronger inflammatory response than KD-alone, but with no differences in cardiac complications. Limited studies have shown the efficacy of IFX in treating KD patients with SARS-CoV-2 infection. Roh et al. (70) found that the number of KD patients with COVID-19 receiving IFX was higher than those KD patients without a history of COVID-19 (two patients vs none), but it was not statistically significant. Therefore, the exact roles of IFX in treating KD patients with COVID-19 and patients with MIS-C still need further investigation.

## 9 Conclusion and perspective

This study summarized the current evidence on the efficacy and adverse effects of IFX in treating KD via a comprehensive review (**Table 1**; **Supplementary Figure 2**). The establishment of therapeutic approaches for KD is a major issue, and the choice of treatment and the timing of therapeutic interventions are also important. A better exploration of the pathophysiological mechanisms of KD may help to design and select the best treatment for the sufferers. Although various treatments exist in clinical practice, each treatment has refractory cases and lacks uniformity, which is needed to solve in future. Although KD is commonly a self-limiting illness, mismanaged of this disease may cause several serious complications, such as CALs, thrombosis, myocarditis, pneumonia, and kidney injury. Based on the results from the aforementioned studies, IFX could alleviate or prevent the progression of CALs, but some investigators failed to find a significant reduction of CALs. On the other hand, there are only a few reports showing the efficacy of IFX in treating KD patients with thrombosis, pneumonia, and kidney injury, which is still warranted to be confirmed by more high-quality studies with large samples. It was reported that 10%–20% of patients with KD are resistant to IVIG with aspirin (the first-line therapy for KD). For the IVIG-resistant KD patients, IFX may serve as the second-line therapy. However, if second-line therapy is unsuccessful in treating IVIG-resistant or refractory KD, the optimal choice for the third-line therapy remains unclear, which needs further prospective RCTs to explore and evaluate the efficacy of the additional therapies in refractory KD.

**Table 1:** The characteristics of the included studies.

Study	Country	Sample size	Infliximab Administration	Treatment outcomes or main findings	Adverse events
Weiss et al. 2004 (18)	USA	KD patient with resistant to IVIG and corticosteroids (n=1)	5 mg/kg of infliximab (on Days 45, 59, and 89)	1. No further fever after the first infusion. 2. Pericardial effusion resolved. 3. Inflammatory markers returned to normal. 4. Infliximab was an effective adjuvant therapy in patients refractory to intravenous immunoglobulin or corticosteroids treatments.	NA
Burns et al. 2005 (33)	USA	n=17	A single infusion of 5 or 10 mg/kg of infliximab	1. Cessation of fever occurred in 13 of 16 patients. 2. Infliximab treatment decreased the C-reactive protein (CRP) level. 3. Patients with arthritis treated with 10 mg/kg of infliximab resulted in dramatic and permanent resolution of their arthritis within 12 hours.	There were no adverse reactions or complications of infliximab infusion.
Burns et al., 2008 (49)	USA	RCT: n=24	A single infusion of 5 mg/kg	IFX infusion and IVIG resulted in the cessation of fever within 24h in 11 of 12 and 8 of 12 patients.	No infusion reaction and positive skin test for tuberculosis were observed.
Hirono K et al., 2009 (29)	Japan	n=43	A single infusion of 5 mg/kg of infliximab	Fever subsided immediately after infliximab treatment (18.6%); Serum proinflammatory cytokines declined remarkably upon infliximab treatment; It might be one of the adoptive therapies in refractory KD patients; But infliximab treatment could not completely inhibit local vasculitis.	NA
Song et al. 2010 (36)	Korea	n=16	A single infusion of 5-6.6 mg/kg of infliximab	1. Cessation of fever presented in 13 of 16 patients (81.3%). 2. CRP level decreased in 87.5% of patients. 3. No remarkable further progression of coronary artery (CA) lesions developing after treatment. 4. Two patients with fever and arthritis achieved dramatic and permanent resolution of their arthritis within 12 hours after treatment.	One case with acute hepatitis occurring during treatment followed by calculous cholecystitis 4 months later.
Shirley et al., 2010 (32)	USA	n=1	Intravenous infliximab with 5 mg/kg	1. Decreased CRP. 2. No abnormalities of the coronary arteries. 3. Infliximab and prednisolone could serve as the first line therapy for KD. 4. The patient defervesced quickly and tolerated with IFX treatment.	The coronary arteries were normal. Without any further adverse events.
Son et al., 2011 (22)	USA	RCT: n=106	A single infusion of 5 mg/kg	1. Fewer days of fever was observed under IFX treatment than the control group (median 8 days vs 10 days, P=0.028). 2. Shorter lengths of hospitalization were observed in the IFX infusion group (median 5.5 days vs 6 days, P =0.04).	1. IFX and the placebo group did not differ dramatically in adverse events (0% vs 2.3%, P =1.0). 2. No symptoms of congestive heart failure was identified in any patient with IFX treatment. No patient had an ejection fraction <60% after IFX treatment. 3. However, IFX could not improve coronary artery outcomes. No AEs related to IFX treatment were detected during the follow-up.
Dogra et al., 2013 (37)	India	n=1	A single infusion of 5 mg/kg	An 18-mo-old girl who presented with features of incomplete KD and was refractory to IVIG and IFX.	This patient was response to pulse intravenous methylprednisolone therapy.
Sivakumar et al., 2013 (39)	India	n=1	NA	Remarkable reduction in thrombus size and the size of the right coronary artery aneurysm.	Repeated fever and thereby necessitating prolonged oral steroid use.
Tremoulet et al., 2014 (11)	USA	RCT: n=196	A single infusion of 5 mg/kg of infliximab	Compared with the placebo group, patients treated with infliximab had fewer days of fever (p<0.0001). Infliximab-treated patients had greater reductions in erythrocyte sedimentation rate (p=0.009), Z score of the left anterior descending artery (p=0·045), CRP (p=0.0003), and in absolute neutrophil count (p=0.024).	No serious adverse events were directly attributable to infliximab.
Ogihara et al. 2014 (30)	Japan	n=8	5 mg/kg of infliximab	Infliximab treatment was corelated to both the signaling cascades of KD inflammation and multiple transcripts related to intravenous immunoglobulin (IVIG) resistance factors.	NA
Sonoda et al., 2014 (44)	Japan	n=76	A single infusion of 5 mg/kg	1. IFX improved the fever and conjunctival injection of KD patients. 2. IFX treatment reduced fever to 37.5°Cin 70 of 76 cases (92.1%). 81.6% of the cases presented with a rapid declination of fever within 2d after IFX administration without any recurrence. 3. IFX could decrease the inflammatory cytokines. 4. Clinical symptoms and blood laboratory data were also improved in these cases.	1. Seven patients (9.2%) showed drug eruptions and liver dysfunction. 2. Four patients (5.3%) suffered from infectious diseases with bacteremia, phlebitis, and urinary tract infections. 3. Eight patients (10.5%) emerged with arthritis. 4. No severe life-threatening adverse event was observed.
Singh et al.2016 (61)	India	n=23	A dose of IFX was 5–7 mg/kg given intravenously.	1. Patients under IFX treatment responded promptly with resolution of fever (11/23). 2. One patient who did not respond to IFX (1/23). 3. Nine severe KD patients with CAAs. No further progression of CAAs was detected among the two patients (2/9).	1. No adverse reactions were observed during the infusion of IFX. 2. None of these patients developed tuberculosis or any other significant infection in the follow-up. 3.One patient (1/23) developed an acute febrile illness two weeks after IFX treatment which responded well to antimicrobials.
Youn et al., 2016 (34)	Korea	KD patients with resistant to IVIG (n=43)	A single infusion of 5 mg/kg	1. Infliximab treatment resulted in shorter duration of fever and fewer days of hospitalization. 2. Treatment resistance was lowere in the infliximab group than in the IVIG group (9.1% vs. 34.4%, *P* = 0.093). 3. The median duration of fever was 6 hours in the infliximab group and 17 hours in the IVIG group (*P* = 0.044). Defervescence occurred within 24 hours in 9 of the 11 subjects (81.8%) treated with infliximab and in 18 of the 32 subjects (56.3%) retreated with IVIG (*P* = 0.098). The median days of hospitalization were short in the infliximab group than the IVIG group (8 days vs 10 days, *P* = 0.046). Coronary artery lesions (CALs) presented in only 1 subject (9.1%) retreated with infliximab, but in 4 of the 32 subjects (12.5%) retreated with IVIG (*P* = 0.411).	1. One subject (9.1%) experienced skin rash developed during infusion of infliximab. 2. No serious AEs such as anaphylactoid reaction, severe infections, transient hepatomegaly, liver function, depressed myocardial contractility, or heart failure were observed.
Han et al.2018 (48)	China	RCT: n=154	A single infusion of 5 mg/kg	1. Fewer refractory KD patients were detected in the combined treatment group than in the IVIG group (4 *vs.* 14, *p*<0.001). 2. IFX treatment had a better outcome with shorter fever durations, hospital stays, and coronary artery dilation. 3. A better anti-inflammation effect was observed. 4. There was no significant differences in the incidence rate of coronary artery aneurysms between the 2 groups (*p*>0.05).	NA
Jone et al., 2018 (52)	USA	n=69	A single infusion of 5 mg/kg	IFX combined with IVIG as initial therapy reduced the need for additional therapy in KD patients with CALs. In addition, this combination also reduced the length of hospital stay.	One patient presented with infusion reaction in IXF plus IVIG (1/35).
Mori et al., 2018 (16)	Japan	RCT: n=31	A single infusion of 5 mg/kg	Defervescence rate within 48h was greater with IFX than VGIH (76.7% vs 37.0%, p=0.023). Moreover, defervescence was achieved earlier with IFX (p=0.0072).	1. CALs occurred in 1 (6.3%) and 3 (20.0%) patients receiving IFX and VGIH. 2. No serious adverse events were observed in the IFX group. 3. AEs presented in 15/16 (93.8%) and 15/15 (100.0%) patients in the IFX and VGIH groups, respectively. 4. Adverse drug reactions presented in 11/16 (68.8%) and 10/15 (66.7%) in the IFX and VGIH groups, respectively. 5. NO infusion reactions were observed. 6. Other AEs in the IFX group included nasopharyngitis, upper respiratory tract infection, and upper respiratory tract inflammation. 7. CALs presented in one patient receiving IFX (6.3%).
Nakashima et al., 2019 (71)	Japan	n=27	NA	High procalcitonin or low sodium levels independently predicted the resistance of IFX treatment in patients with KD who were refractory to IVIG.	NA
Nagatomo et al., 2018 (8)	Japan	KD patients with CAA, n=49	A single infusion of 5 mg/kg	1. IFX administration significantly reduced the level of CRP. 2. Maximum z-score and response to IFX were independently associated with disease regression.	NA
Hur et al., 2019 (21)	Korea	RCT: n=102	A single infusion of 5 mg/kg	1. Early treatment of IFX reduced the incidence of significant coronary artery aneurysms (CAAs) in subjects with IVIG-resistant KD. 2. IFX treatments resulted in a rapid defervescence and a shorter length of hospitalization. 3. The overall response rate to IFX infusion was 89/102 (87.3%).	Patients with IVIG-resistant KD were well tolerated under IFX treatment. No complications and infusion reactions were observed.
Furuta et al., 2020 (54)	Japan	n=11	A single infusion of 5 mg/kg	Ten patients (91%) presented with defervescence within 48h after IFX infusion.	1. Two patients occurred CALs (18%) after IFX treatment. 2. One patient presented with hyper-triglyceridemia with spontaneous resolution. 3. Two patients developed skin rash. 4. Six patients received BCG vaccination before IFX treatment but none of them developed disseminated BCG infection.
Miura et al., 2020 (46)	Japan	RCT: n=291	A single infusion of 5 mg/kg	1. The fever resolution rate within 48h after IFX infusion was 77.4%. 2. IFX treatment inhibited acute inflammation within 10 days of illness. 3. No increase in size or occurrence of CALs was detected after IFX treatment.	1. Adverse drug reactions (ADRs) were recorded in 12.4% (the most common one was rash); Serious adverse drug reactions were 3.1%. 2. No live vaccine-related infections was detected. 3. The incidence (4.2%) and severity of CALs did not alter significantly. 4. Other ADRs influenza, bronchitis, cellulitis orbital, gastroenteritis, Chlamydia pneumonia, respiratory tract infection, infusion reaction (1.4%), and decreased white blood cell count (0.3%).
Shimada et al., 2020 (42)	Japan	n=17	A single infusion of 5 mg/kg	1.The CRP level and the total number of plasma exchange sessions reduced under IFX treatment. 2. The prognosis of coronary artery disease and complications of plasma exchange did not significantly differ in the IFX group.	NA
Johnson et al., 2021 (50)	USA	n=100	A single infusion of 10 mg/kg	IFX infusion turned out to a shorter length of stay and less hospital costs, regardless of age.	NA
Pilania et al.2021 (40)	India	n=4	A single infusion of 5 -10mg/kg	Four children with KD combined with macrophage activation syndrome (MAS) received IVIG as first-line therapy and then treated with IFX.	There have been no recurrences of MAS in the four patients under IFX treatment, and none of them had any short-term adverse effects.
Mitsui et al., 2022 (56)	Japan	n=1	A single infusion of 5 mg/kg	The fever and other symptoms immediately improved after IFX treatment.	Psoriasiform eruption with arthritis was occurred after IFX administration.
Matsubara et al., 2022 (41)	Japan	n=1	A single infusion of 5 mg/kg	The patient’s fever reduced on the 24th day after treatment with IFX. Arthralgia improved significantly, and the patient could walk on the 26th day.	NA

NA, not available; CALs, coronary artery lesions; IVIG, intravenous immunoglobulin; CAAs, coronary artery aneurysms; VGIH, IV polyethylene glycol-treated human immunoglobulin.

The application of IFX for either initial combination therapy or salvage therapy in KD has been reported in various studies, but there is no clear optimal application protocol. Since IFX is usually administered late in the disease, early detection of IFX resistance is important to reduce CAA. However, there are no biomarkers to predict IFX resistance. Nakashima et al. (71) aimed to develop a clinical prediction model for IFX responsiveness in patients with IVIG-refractory KD. Several studies (72, 73) proposed procalcitonin as a biomarker to predict the severity of KD. However, there is still no information about procalcitonin related to IFX resistance in patients with refractory KD.

The indications for the application of IFX and the optimal timing of its application are not clear and vary widely among different studies in different regions. Most studies have reported that IFX is more effective in reducing fever in children with IVIG or hormone nonresponse, with sensitivity rates ranging from 76.7% to 90.9%. Initial intensive treatment with IFX combined with IVIG is routinely used in children with KD complicated by CAA at 1 medical center in the United States (47), while IFX is used for salvage therapy in Japan (1, 74), and Korea (21). There is relatively little experience with the application of IFX due to most patients are effective on IVIG and high-dose aspirin therapy. And most of the current relevant studies have small sample sizes and lack of relevant controlled studies. Therefore, further large-sample studies are still warranted in the future to find factors associated with IFX insensitivity. Multicenter trials with large sample size and long-term follow-up are needed to evaluate the clinical efficacy and safety of IFX for IVIG-resistant KD or refractory KD.

In the future, we believe there will be a treatment approach that is tailored to the genetic polymorphisms and pathological characteristics of each KD case. A customized treatment strategy with less adverse effects and complications of coronary artery disease can be achieved possibly later. For the potential refractory KD patients with risk factors, intensive primary therapy in these populations will be needed. In addition, close monitoring for cardiac-related complications by frequent echocardiography will be also required.

## Author contributions

JC and JL contributed to conceive and design the study, LX and SZ extracted the data and revised the manuscript. YY wrote the manuscript. All authors contributed to the article and approved the submitted version.

## Supplementary material

Supplementary Figure 1 Flow chart of study selection.

Supplementary Figure 2 The efficacy and adverse effects of IFX in treating KD.
