# Supplementary figures and images for: Current knowledge of TNF-α monoclonal antibody infliximab in treating Kawasaki disease: a comprehensive review
Source: Front Immunol. 2023 Oct 23;14:1237670. doi: 10.3389/fimmu.2023.1237670 (PMC10626541; doi:10.3389/fimmu.2023.1237670)

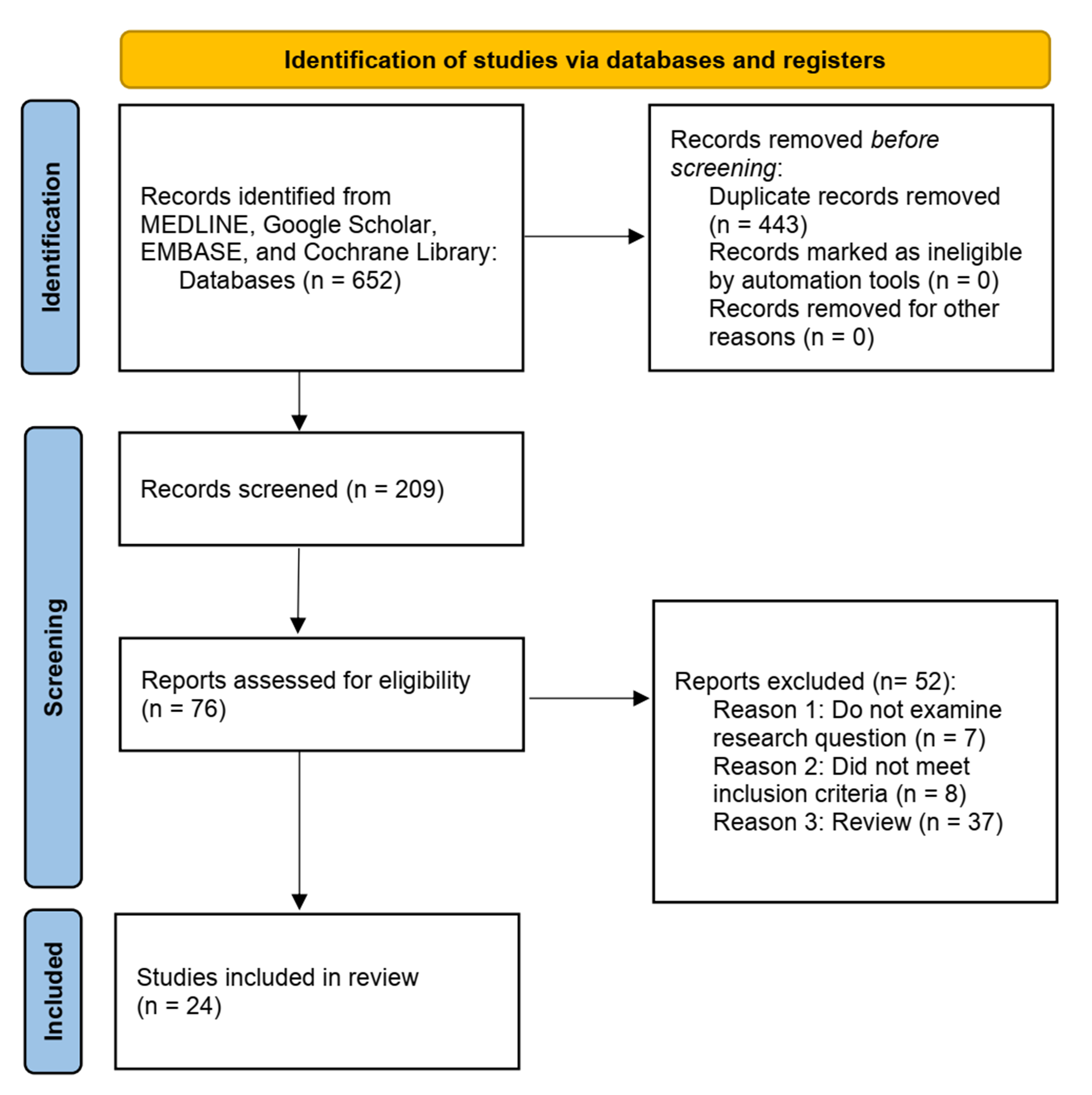

Supplement: Supplementary Figure 1 — Flow chart of study selection. [file Image_1.tif]

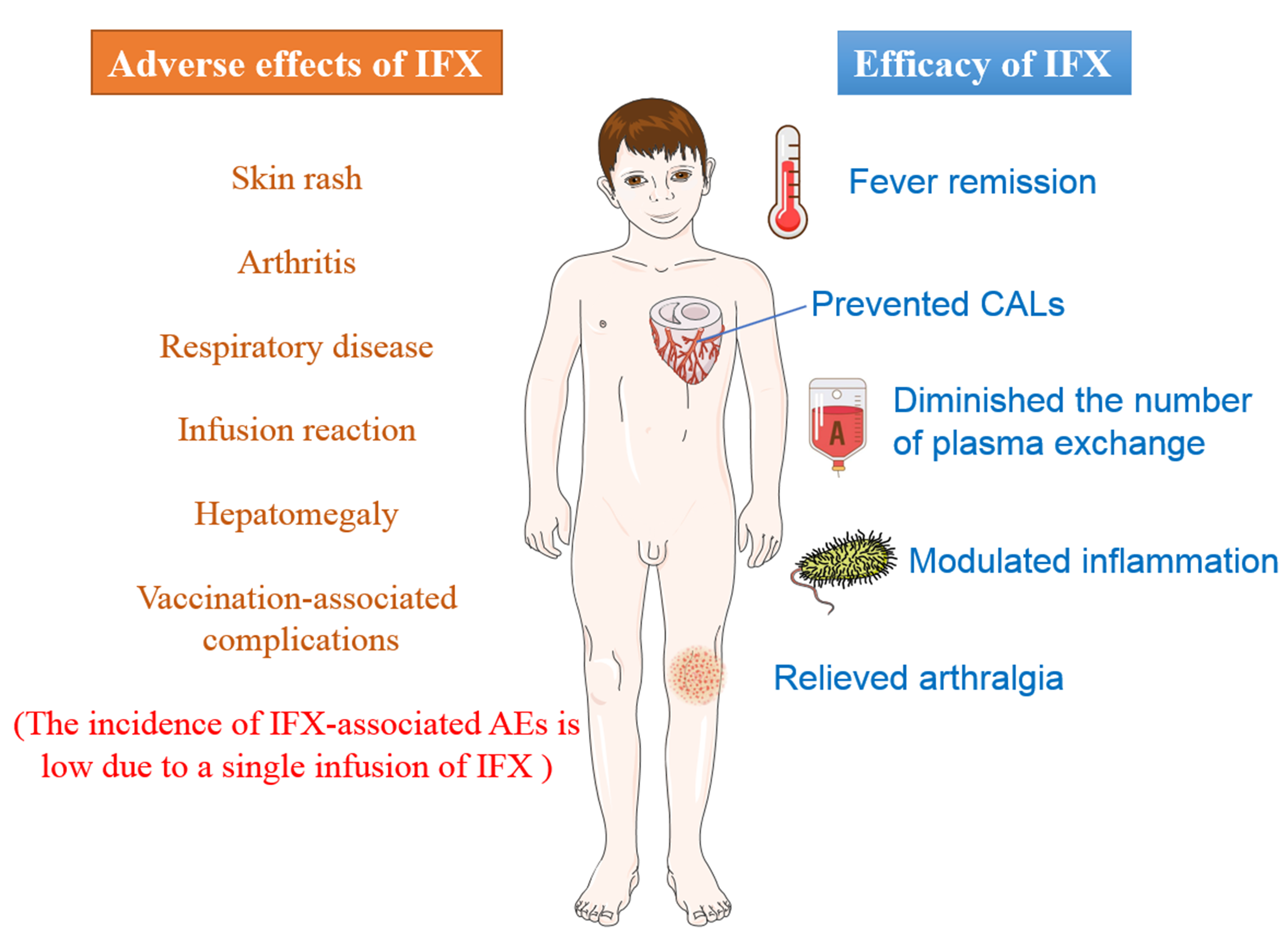

Supplement: Supplementary Figure 2 — The efficacy and adverse effects of IFX in treating KD. [file Image_2.tif]
